# Medical Students’ Self-Perceptions of Harassment During Clinical Placement

**DOI:** 10.1007/s40670-023-01926-5

**Published:** 2023-10-27

**Authors:** Marcus A. Henning, Josephine Stonyer, Yan Chen, Benjamin Alsop-ten Hove, Fiona Moir, Ties Coomber, Craig S. Webster

**Affiliations:** 1 Centre for Medical and Health Sciences Education, University of Auckland, Building 507, Grafton, Auckland, 1023 New Zealand; 2 School of Medicine, University of Auckland, Grafton, Auckland, 1023 New Zealand; 3 Department of General Practice and Primary Healthcare, Population Health, University of Auckland, Grafton, Auckland, 1023 New Zealand

**Keywords:** Workplace learning, Medical students, Harassment, Clinical placement

## Abstract

**Objectives:**

Exploring workplace dynamics during clinical placement is crucial to determine whether medical students are encountering safe and meaningful learning experiences. The aim of this original article is to describe medical students’ reported harassment experiences whilst on clinical placement.

**Design:**

Medical students in years 4 to 6 were invited to participate in the survey. In this mixed-methods study, data collection included demographic information, responses to the Generalized Workplace Harassment Questionnaire, and qualitative commentaries.

**Results:**

Two hundred and five students completed the questionnaire. Medical students experienced harassment in areas of verbal aggression, disrespect, isolation/exclusion, threats/bribes, and physical aggression. Concerning levels of occurrence were noted for disrespect, isolation/exclusion, and verbal aggression.

**Conclusions:**

Many medical students in this study reported experiencing harassment during their clinical placements indicating that harassment during clinical placement continues to be of concern in medical education. The findings indicate that further initiatives need to be designed to identify and respond to these cases of workplace harassment and that power imbalance and safe reporting appear to be further issues of concern. It was evident that students need to feel safe enough to be able to report harassment experiences to allow managers and educators to address the full extent of the problem.

## Background

There is growing evidence indicating that harassment amongst medical students is widespread, under reported and a historical and prevailing issue [1]. The exploration of harassment experiences of medical students is an important and developing area of research given the powerful and lasting effects of harassment and its influence on career choice [2, 3]. In addition, medical students require training in clinical settings, which may put them at risk of being exposed to forms of mistreatment or harassment in the workplace [4]. Harassment in the hospital setting has been defined as any, “unreasonable, unwelcome comment or behaviour that offends, humiliates, or intimidates. The behaviour is either repeated or a serious, one-off incident that has a negative effect on safety, health, performance or job satisfaction” [5]. In many countries, workplaces are legally obliged to provide environments that are physically and psychologically safe [6]. In this study, we were interested in determining the extent to which medical students experienced harassment during their clinical training within healthcare workplace settings.

Harassment can be linked to discrimination based on sex, gender, sexual orientation, race, religion, physical characteristics, or mental ability [7, 8]. Occurrences of harassment within higher education settings have been reported in areas of sexual harassment, mobbing, racial and gender bullying, and social exclusion [9]. As a point of explanation, mobbing is a term used to describe unfair treatment, through frequent and systematic harassment, of one person in a workplace by group of powerful personnel, often resulting in social isolation and expulsion from the organisation [10]. In a recent systematic review of academic bullying in medical settings [11], the authors reported that overall bullying behaviours can be categorised as creating instability within the work environment (destabilisation), threats to professional status (e.g. excessive monitoring and criticism), isolation (e.g. social and professional exclusion), and overwork (e.g. undue pressure to produce work). The study sample included preclinical medical students, who were reported as being at greater risk than residents and consultants of experiencing persistent criticism, who were then often more prone to psychiatric distress. It was further noted that most of the perpetrators of students were consultants.

Harassment behaviours reported in reference to the medical education context are commonly cited and include sexual harassment, physical and/or psychological abuse, mobbing, and discrimination based on age, race, gender, and sexual preference [2, 3]. In their study, Mavis and colleagues [12] state that between 17 and 20% of medical students in the USA reported experiencing some form of mistreatment. Examples of mistreatment include being publicly belittled or humiliated, along with being called sexist names or similar remarks, being on the receiving end of racially or ethnically offensive remarks, and being asked to perform personal services. Nonetheless, one scoping review concluded that the actual prevalence of harassment being experienced by medical students within the New Zealand clinical context is not well documented [2].

There are numerous instruments that could be used to assess levels of harassment in the clinical context. In their review article, Henning and colleagues [9] have provided a list of questionnaires that can be used to measure the incidence and levels of harassment in higher education settings. These included the questionnaires that specifically measured sexual harassment, attitudes to women, and mobbing in the nursing context. In addition, other instruments focussed more on generalised workplace abuse and bullying. In this study, we were interested in determining the extent to which medical students experienced generalised workplace harassment during their clinical training within healthcare workplace settings. Therefore, the aim of this paper is to describe medical students’ reported harassment experiences whilst on clinical placement. The two research questions are:

1. What type of harassment experience, if any, did medical students encounter whilst on clinical placement?
2. What are the differences in harassment experiences in terms of medical students’ age, sex, ethnicity, or year of study?

## Materials and Methods

### Study Design

This is a mixed-methods study integrating descriptive information from a cross-sectional survey and students’ comments about their experience of harassment during clinical training. Therefore, the qualitative comments were employed to build on the descriptive numerical data [13].

### Participants

All students (*N* = 810) studying in the 4th, 5th, and 6th years of the Bachelor of Medicine and Bachelor of Surgery (MBChB) programme at the University of Auckland (2019) were invited to contribute to this survey. We selected medical students during years 4 to 6 because they are required to attend clinical placements, which are designed to ensure practice-based learning as supervised by a qualified clinician, but which is also known to be a workplace setting where harassment may occur. The total number of students enrolled in the 4th year was 279, 5th year 266, and 6th year 265. We estimated that to achieve a margin of error for our survey at 6% at the 95% level of confidence, which is within the 8% margin of error considered acceptable for generalisable commercial surveys [14], we would require more than 201 responses [15].

### Procedure

Ethics approval for the implementation of the study was sought and obtained from the University of Auckland Human Participants Ethics Committee (Ref. 023525).

Participation was voluntary and students were informed about the study by email by one of their student peers, who is a co-author (JS) of this paper, on 11th November 2019. This initial communication was followed by two reminder emails at the end of November and beginning of December (2019). Data were collected over a 1-month period and given this was a cross-sectional survey, students were instructed to respond to the survey at one time point only. In addition, a monetary incentive was provided whereby a draw for one of five $NZ100 vouchers was provided although students did need to provide their contact details to be eligible for this. This provision of contact details was not linked to their anonymised responses to the questionnaire. The anonymous survey was sent to students prior to final completion of courses for that semester, but after all grades had been released. Students were asked to participate, outside of scheduled class time, using Google Forms [16].

### Measures

A background questionnaire was developed eliciting the following information: (1) age (years); (2) sex (male, female, other); (3) ethnicity (based on the New Zealand census format) [17]; and year of study (4, 5, or 6). Ethnicity was transformed into categories consistent with the Ministry of Health prioritisation protocol [18]. An open-ended commentary box was also provided at the end of the questionnaire to enable students to describe any issues associated with harassment they may have experienced.

Due to the workplace focus of the study, we used the work of Rospenda and Richman [19] as our guide for exploring the incidence and types of potential harassment behaviours that students may have experienced. Therefore, an adapted version of the Generalized Workplace Harassment Questionnaire (GWHQ) was employed [19]. This questionnaire measures generalised workplace harassment, which “constitutes interpersonally hostile interactions such as being sworn at, subjected to humiliating or demeaning behavior, threatened, or otherwise mistreated in the workplace” [19].

This questionnaire contained the original 29 items measuring issues related to workplace harassment and aggression. Given the descriptive nature of this study, we included all the items in our analysis and, therefore, summarised the items according to the five GWHQ conceptual factors, namely verbal aggression (9 items), disrespect (9 items), isolation/exclusion (5 items), threats/bribes (3 items), and physical aggression (3 items) [19]. All items incorporated a 5-point Likert scale (1 = Never to 5 = Very often), with a higher score indicating a higher incidence of that harassment behaviour. In the original questionnaire, the authors used a 3-point rating scale (1 = Never, 2 = Once, 3 = More than once) [19].

Rospenda and Richman [19] provided information that showed good reliability over three time periods (time 1 = 0.92, time 2 = 0.91, time 3 = 0.92), with reasonable reliabilities for the four extracted factors (verbal hostility = 0.88; covert hostility = 0.78; manipulation = 0.58; physical hostility = 0.51) which represented 25 of the 29 items. However, given the exploratory mixed-methods nature of this study, we wanted to utilise all the 29 items to investigate the full range of possible responses and thus opted to employ the conceptual factors, which were developed using content validity methods. We rationalised that the full 29 items would allow us to highlight the richness of the responses in more depth. The five conceptual factors were denoted as follows: verbal aggression, disrespect, isolation/exclusion, threats/bribes, and physical aggression [19]. We acknowledge that this gave this study a more qualitative emphasis but felt that this would be a valuable perspective to consider.

### Data Analysis

First, we assessed the demographic data to ensure the sample was representative of the wider population. Second, we generated descriptive statistics that summarised the five conceptual factors developed by Rospenda and Richman [19]. We also aligned an exemplar student quote, taken from the qualitative section of the survey, that was the most meaningful in content to each of Rosenda and Richman’s five conceptual factors [19]. Third, we computed descriptive statistics for the GWHQ items [19]. Last, using IBM SPSS version 26 [20], we computed a series of multivariate tests to evaluate the presence of any discerning significant results among the demographics variables (age, sex, ethnicity, and year of study).

## Results

### Response Rate and Participant Data

A total of 205 students (response rate = 25%) completed the online survey (comprising a 6% margin of error at the 95% confidence level). As shown in Table 1, more female students (68.8%) than male students responded to the survey. We obtained responses across all ethnic groupings, suggesting that the response groups would likely be reasonably representative of the wider group. The average age of participants was 23.75 years (SD = 2.91, max = 38 years, min = 20 years). We were unable to access data regarding the cross tabulations between age, sex, ethnicity, and year of study.

**Table 1:** Sociodemographic characteristics of the study sample (*n* = 205)

Characteristics	Frequency	%
Sex		
Male	63	30.7
Female	141	68.8
Other	1	0.5
Year of study		
4	83	40.5
5	62	30.2
6	60	29.3
Ethnicity		
Māori	20	9.8
Pacific Islands	13	6.3
Asian	70	34.1
New Zealand European	90	43.9
Other	12	5.9
	Mean	SD
Age	23.75	2.91

### Scale Analysis

In reference to Table 2, internal consistency coefficients computed for each of the scales and four of the five scales were in the satisfactory range [21]. However, the score for Threats and Bribes was indicative of poor internal consistency, and thus, this factor was not incorporated in the multivariate sub-group analyses.

**Table 2:** The five GWHQ conceptual factors: Descriptive statistics, Cronbach alpha scores and exemplar quote

**Scale**	Mean	SD	Cronbach *α*	Exemplars from students’ comments (one comment per table row)
Disrespect	1.68	0.60	.84	It is difficult to have autonomy over your life as you are completely at the whim of the medical school and your hospital team. This is in turn makes it difficult to plan anything as every 4–6 weeks your timetable completely changes, and you have no control over this
Isolation/exclusion	1.48	0.50	.62	If you are consistently ignored it can be hugely demoralising. I’d honestly rather be yelled or made fun of than ignored. I can handle someone yelling at me, and I can turn a joke and laugh at myself. But there’s not much I can do when the consultant or registrar won’t even talk to you or just walks off without you. There have been some runs where I’m like “can they see me?” “am I invisible today?”
Physical aggression	1.04	0.19	.58	When I was in 4th year I was bullied and tormented by a junior doctor, with whom I had a previous complicated relationship with I was terrified to go forward and complain to the faculty because • I didn’t think I would be believed • They had threatened me physically in the past • They had pictures and text messages of/from me that were compromising that I think they would have shared • this Dr was friends with a lot of the other junior doctors • I was terrified of the repercussions from them
Threats/bribes	1.26	0.36	.24	Every day we are forced to go somewhere where you are not wanted and no one has time for you. Further, we have to stay there even when there is nothing to learn because leaving to go study makes us look bad
Verbal aggression	1.60	0.53	.82	I watch, and experience, so many medical students belittled on a daily basis. I’ve also seen and heard some incredibly sexist and misogynistic things said to female students

The mean scores (1.04 to 1.68) for each of the scales indicated relatively low levels of perceived experience of harassment. Consistent with the relatively low rate of reported harassment, only a few students provided written comments contextualising these events (*n* = 14, 7%) in the open-ended commentary box. Nonetheless, these quotes describe serious and concerning harassment experiences during clinical placement in considerable detail with the potential to substantially undermine the quality of a student’s educational experience and affect career choices (Table 2). Hence, we consider these to be critical harassment events, in the sense that even one instance of such events warrants serious consideration [22]. Therefore, we have assigned an exemplar quotation to each of the GWHQ conceptual factors in order to better contextualise the quantitative results (Table 2).

### Item Analysis

Table 3 provides descriptive data in relation to each of the items in the GWHQ. Certain items appear to highlight issues of concern (mean scores > 2). The score of “2” was used as a cutoff measure because it indicated that some experience of harassment had occurred. According to this criterion, highlighted scales and items of concern were:

1. Disrespect, i.e. talked down to you (e.g. treated you like a child or as inferior to them)? And treated you or evaluated you as though you were less good at your work than you really are? And asked you to do work which really wasn’t part of your job?
2. Isolation/exclusion, i.e. ignored you or your work contributions?
3. Verbal aggression, i.e. prevented you from expressing yourself by interrupting you? Or told you insulting jokes?

**Table 3:** Items (according to the five GWHQ conceptual factors) with distributions and descriptive measures

Scale	Item	Response options					Mean	SD
		**1 = never**	**2**	**3**	**4**	**5 = very often**		
Disrespect	Humiliated or belittled you in front of others?	83	80	31	10	1	1.86	0.88
	**Talked down to you (e.g., treated you like a child or as inferior to them)?**	60	61	45	25	14	2.38	1.22
	**Treated you or evaluated you as though you were less good at your work than you really are?**	81	63	35	18	8	2.07	1.13
	Expected less of you than others in your position?	145	37	17	3	3	1.45	0.82
	Tried to control your non-work related time or activities?	151	29	16	6	3	1.44	0.87
	Left notes, signs, or other materials which were meant to embarrass you?	200	4	1			1.03	0.20
	Treated you unfairly compared to others in your some position (e.g., in terms of tasks or assignments, salary, promotions, resources, reprimands)?	147	38	12	4	4	1.44	0.85
	**Asked you to do work which really wasn’t part of your job?**	77	59	46	16	7	2.11	1.10
	Blamed you personally for things that other people did, or that weren’t your fault?	149	38	13	4	1	1.39	0.74
Isolation/exclusion	Labelled you a “troublemaker” if you expressed a difference of opinion?	176	16	8	4	1	1.23	0.66
	Took credit for your work or ideas?	137	48	16	3	1	1.45	0.74
	**Ignored you or your work contributions?**	74	61	42	19	9	2.16	1.15
	Turned others in your work environment against you?	182	16	3	3	1	1.17	0.56
	Excluded you from important work activities or meetings?	153	35	11	3	3	1.38	0.78
Physical Aggression	Pushed you or grabbed you?	195	7	2	1		1.07	0.34
	Threw something at you?	202	1	1	1		1.03	0.26
	Hit you physically?	203	2				1.01	0.10
Threats/bribes	Pressured you to change your beliefs or opinions at work?	111	55	26	12	1	1.72	0.93
	Offered you a subtle or obvious bribe to do something that you did not agree with?	199	4	2			1.04	0.24
	Threatened that they would “get back at you” if you resisted doing something that you thought was wrong, or if you challenged things about the workplace?	200	3	2			1.03	0.23
Verbal Aggression	Yelled or screamed at you?	138	50	14	3		1.42	0.69
	Gossiped about you and/or spread rumours about you behind your back?	118	55	24	6	2	1.63	0.87
	**Made negative comments to you about your intelligence, competence, or productivity?**	66	78	42	14	5	2.09	1.01
	Made hostile or offensive gestures at you?	161	33	9	2		1.28	0.59
	**Prevented you from expressing yourself by interrupting you?**	80	62	45	14	4	2.02	1.03
	Swore at you?	178	20	5	2		1.18	0.50
	Made negative comments to you about your personality?	141	38	17	6	3	1.50	0.88
	**Told you insulting jokes?**	88	51	43	17	6	2.03	1.11
	Made negative comments to you about your appearance?	167	28	7	2	1	1.25	0.61

Items in bold are of particular concern, as indicated by mean score > 2

Overall, the item distributions revealed that students in this sample had experienced some form of harassment in all the scales appraised. We conducted a frequency check of the data to gauge the percentage of those students who had experienced some form of harassment (indicated by a value greater than 1), albeit likely at a low level of occurrence. The percentages of students reporting that they had encountered some form of harassment were as follows: disrespect (89%), isolation or exclusion (72%), threats or bribes (47%), physical aggression (6%), and verbal aggression (87%). While this is of concern, all mean scores and distributions indicated low levels of reported occurrence.

### Sub-Group Analysis

A series of multivariate analyses of variance (with measures of disrespect, isolation/exclusion, physical aggression, and verbal aggression) were conducted on sub-groups. No significant differences were noted for sex, ethnicity, and year of study. However, age was found to be significantly correlated with four of the five scale measures.

More specifically, the Wilks’ lambda test statistic from multivariate analyses indicated no significant differences for sex (*Wilks’ lambda* = 0.99, *F*(3, 200) = 0.21, *p* = 0.85); ethnicity (*Wilks’ lambda* = 0.92, *F*(16, 602) = 0.99, *p* = 0.47); and year of study (*Wilks’ lambda* = 0.98, *F*(8, 398) = 0.58, *p* = 0.79). Nevertheless, age was found to be positively and significantly correlated with disrespect (*r*(202) = 0.29, *p* < 0.001), isolation/exclusion (*r*(202) = 0.18, *p* = 0.01), and verbal aggression (*r*(202) = 0.18, *p* = 0.008), although no significant correlation was found with physical aggression (*r*(202) = 0.02 *p* = 0.81).

## Discussion

This cross-sectional mixed-methods study provided self-reported information on medical students’ experiences of harassment during their clinical training. It is clear from the findings in this study that many students in this sample had reported encountering some form of harassment in the areas of disrespect, isolation or exclusion, threats or bribes, and verbal aggression during their clinical placement, which ranged from 47 to 89%. Only physical aggression indicated a low level of experience at 6%. However, the sub-group analysis suggested there were no discernible differences in terms of sex, ethnicity, or year of study. Nevertheless, older students tended to report experiencing higher levels of harassment compared to younger students. There was no discernible reason for this given that some studies have reported that older students tend to report harassment less often [23]. One hypothesis is that the discrepancy relates to the willingness to report rather than the experience of more harassment and this could be linked to the idea of power, which will be subsequently discussed.

We will now consider each of the conceptual factors described by Rospenda and Richman [19] in more detail.

### Disrespect

Rospenda and Richman [19] propose that this factor underscores associations with humiliation, belittlement, feeling inferior, unfair judgment and treatment, controlling behaviours, embarrassment, inequitable task allocation, and unwarranted blaming. The concept of disrespect overlaps with incivility, or being devalued or treated as less capable [24]. The findings in this study indicated that some students experienced persistent disrespectful behaviours, with three questionnaire items highlighting students’ feeling that they were talked down to (7%), treated as being less good (4%), or asked to do work which they perceived was not part of their job (3%).

One student (Table 2) saw disrespect as linked to a lack of involvement when tasks were being allocated, and thus feeling a lack of autonomy. Leape and colleagues [25] proposed that disrespect can be attributed to several sources and occur at many levels. For example, they cite the problem of learning through humiliation, when senior physicians model disdain when responding to patient questions. Disrespect can be linked to disruptive behaviour resulting in poorer health outcomes for patients [8]. For example, they cite a culture of education through humiliation, and senior physicians modelling of disdain when answering patients’ questions. Disrespect can occur at subtle levels by displaying passive-aggressive behaviours, such as not following through on previously agreed proposals. Disrespectful behaviours can be dysfunctional and entrenched within workplace cultural norms. This form of entrenchment may create an unprofessional hierarchy whereby healthcare workers are perceived as being more valuable than students and patients. Lastly, disrespectful behaviours can stem from the individual (e.g. personality) or environmental (e.g. cultural norms) factors. Leape highlights that students are particularly at risk of experiencing forms of disrespect, including belittlement which was of particular concern in our findings.

### Isolation or Exclusion

Rospenda and Richman [19] contend that this factor is linked to the concepts of being excluded from important conversations or meetings, being used by those in higher positions, having contributions plagiarised, and creating discord in the workspace. The findings indicated that a critical mass of students in this study experienced aspects of being isolated or excluded, for example, 64% of students had specifically experienced being ignored in reference to their work contributions. The quote in Table 2 clearly describes one student’s feelings regarding this type of occurrence (e.g. “am I invisible today?”). The feeling of being excluded or isolated can adversely impact a student’s sense of being a team member culminating in a perception that they are not making a meaningful contribution.

Romanski and colleagues [26] put forward the term the “invisible student” that aptly describes this felt phenomenon. They report students describing this type of treatment as often being covert, whilst stating that it obstructs learning and reinforces their low position in the team hierarchy. The nature of exclusion can also be perceived as being passive, although the impact may have an adverse effect on students’ development, morale, and ultimately their career choices. The converse also appears to be true, that is, that feeling part of the team during clinical placements is likely to increase the chance that students will choose to practice in that area once fully qualified [27].

Some recent literature has focussed on finding ways to meaningfully involve students during clinical training. Ooi and colleagues [28] reported ways in which students could be included when discussing patient issues in handover, such as making time for opportunistic teaching moments. In this way, students could feel valued and part of the team they belong to. In addition, the University of Auckland have put in place an anonymous survey whereby medical students can report experienced or witnessed behaviour associated with bullying, harassment, discrimination, and exclusion [29]. Even though this method may not capture unique instances of harassment, it can isolate clinical settings where students feel unsafe.

### Physical Aggression

Physical aggression is a particularly worrying aspect of harassment with potential legal ramifications if reported. Rospenda and Richman [19] have defined physical aggression in terms of being pushed, grabbed, and physically assaulted. In this survey, we noted that ten students (5%) had reported being pushed or grabbed, three (1%) had reported something being thrown at them, and two (1%) had reported being hit physically — while low rates in absolute terms, these are unacceptably events. It is worrying that there are self-reported instances of this type as this represents one of the most extreme forms of harassment. The instance described by the student in Table 2 is very concerning given that this student was junior and felt unable to report the incident. The feeling of being unsafe and unsupported is a concern for any university or healthcare setting, suggesting that it is crucial to have mechanisms that optimise access to support and safe reporting systems.

Physical aggression is clearly a very explicit form of mistreatment and does occur in healthcare workplaces. Warshawski [30] suggested that many healthcare workers at some time in their career do experience physical assault, although they do not specify the perpetrators. In a further study, the authors stated that 18% of their sample of Nigerian medical students had experienced being slapped, pushed, kicked, or hit [3]. Nonetheless, as with this study, physical assault is typically reported as occurring less often than other forms of harassment. One of the important issues to consider is ensuring that students feel safe and empowered to report instances of physical assault. Students need to be provided with information at the beginning of, or prior to, their clinical rotations regarding this [30]. Reporting systems need to be shown to be effective; otherwise, students may perceive it to be harmful to their future careers or personal safety.

### Threats or Bribes

Rospenda and Richman [19] underscore this aspect of harassment in terms of feeling pressured to change one’s belief or opinion, being subjected to bribes, or being threatened. Students in this study revealed encountering certain harassment behaviours, such as feeling pressured to change one’s beliefs or ideas (46%), reporting of feeling bribed (3%), or feeling explicitly threatened (2%). Although none of these items met the mean score > 2 threshold for highlighting concerning items, 46% of respondents scored 2 or greater for feeling pressured to change one’s beliefs. The notion of feeling pressured to change one’s belief or idea is alarming, particularly given the example provided by the student in Table 2. However, the context of the experience needs to be fully investigated, given that in medicine ideas and beliefs about practice are often challenged, which is part of the ongoing process of being a competent and safe health professional.

In one study, Owaoje and colleagues [3] stated that 26% of their sample of medical students experienced threats of harm, which is demonstrably higher than the reports in this study. The source of these threats appeared to originate from those in power (e.g. consultants, lecturers, and registrars). Issues of power and hierarchy have been highlighted in the literature and often used to explain why some groups may be at risk of bullying [31, 32]. The issue of power is conditional, in that negative use of power can lead to evident cases of mistreatment while positive power dynamics can create constructive learning outcomes [32]. Moreover, the influence of power can occur in both the defined and hidden curricula [32].

### Verbal Aggression

Lastly, verbal aggression is an area often cited as being problematic in the healthcare workplace [33]. Rospenda and Richman [19] have defined verbal aggression in terms of certain behavioural instances, such as being yelled at, sworn at, insulted, and spreading unpleasant rumours. Three items with this scale were highlighted as being problematic, namely experiencing negative comments about performance and intelligence (70%), preventing the expression of one’s views (62%), and being told insulting jokes (59%). The exemplar student quote (Table 2) regarding their experience of verbal harassment is concerning. These experiences and their relatively high level of incidence may be linked to the notion of accepted cultural practices within teams, suggesting staff development in healthcare learning settings is crucial to minimising the occurrence of these modes of communication [8]. Nonetheless, negative comments are likely dependent on the context in which they are given and the use of language underlying the negative comments (e.g. use of personalised demeaning phrases).

Verbal harassment is clearly linked to workplace culture, especially when considering the responses in this survey and those examples found in the wider literature [2, 34]. The cultural norms surrounding ways of communicating can occur at all levels and are often found within the hidden curriculum. As with previous assertions, it is likely to be associated with notions of power and hierarchy. Undesirable consequences can result, such as becoming adversely socialised or using toxic behaviours when communicating with patients and future colleagues [34]. Therefore, the strategies put forward by Kassebaum and Cutler [34] are likely to be still valid today, such as promoting respectful communication at all levels of the learning spectrum, and ensuring methods of communication are audited at both formal and informal levels.

### Limitations

Our response rate of 25% may be perceived as a limitation. However, this yielded a margin of error for our survey of only 6% at the 95% level of confidence, well within the 8% margin of error considered acceptable for generalisable commercial surveys [14]. In addition, response rates are typically low in these types of survey studies aiming to obtain data in this topic area [35]. Fosnacht and colleagues [36] acknowledge that variation exists with defining nonresponse bias, but state that “low response rates may or may not lead to nonresponse bias because answers to survey items may not differ substantially between responders and nonresponders”. In their study, using simulated data, they found that simulated datasets that yielded response rates of 5% generated meaningful correlation coefficients (0.64 to 0.89) with full sample estimates. Therefore, they question the assumption that low response rates are symptomatic of low data quality and further state that response rates of 20 to 25% are likely to yield meaningful survey data.

Moreover, Fan and Yan [37] state that using online survey has several advantages when compared with traditional paper-based survey, such as being more accessible to those participants who have internet access, created quicker response time, lower costs in delivery, and easier data entry options. Nonetheless, Fan and Yan also note that online surveys can be problematic in reaching participants who do not have internet access and often generate low response rates. In reference to the present cohort, lack of internet access is unlikely to be an issue, although the sensitive nature of the questionnaire and students’ high study workload may have impeded students’ willingness to respond to the survey. Further factors that may have obstructed a willingness to respond relate to the sponsor of the survey and the time it would take participants to fill in the questionnaire. The sponsor of this current questionnaire was the university that manages the clinical placement, which may have affected the response rate. Nonetheless, employing a student researcher to orchestrate the communication protocol and data collection of the survey may have mitigated some of the risks associated with this factor. In addition, a monetary incentive was provided to students. The GHWQ has 29 items which may have deterred some students due to their busy schedules, and given that the university medical curriculum requires not only examination commitments but also evaluations of courses and placements beyond the current study. This may have hampered students’ enthusiasm to fill in a survey occurring outside their curriculum duties, even though this survey was explicitly sanctioned by this university.

A related issue that needs to be acknowledged is the potential presence of reporting bias, which may result in the under-reporting or overestimation of incidence [38]. To further validate the data presented in this study would require ongoing studies to audit the potential presence of harassment in clinical settings as experienced by medical students. Nonetheless, occurrences of harassment are evident within this sample establishing it is an issue worthy of further investigation. In addition, as aforementioned, Fosnacht and colleagues [36] have provided a convincing argument to suggest this may not be a critical problem in these types of studies.

Given this is a convenience sample, we made an assumption that the sample participating in this study had representation from key demographic groups (age, sex, ethnicity, and year of study). However, we acknowledge that the sample may not be statistically equivalent in proportionality to the population. We argue that the cross tabulations between age, sex, ethnicity, and year of study would be interesting, but would unlikely add value to the central thesis of the study, which is a mixed-methods approach aimed at exploring and identifying issues of harassment within the clinical context. However, cross tabulations could be scope for further research, although gaining statistical representation at this fine-grained level would likely be very difficult to achieve, especially when measuring this sensitive issue which can only be practically conducted using convenience sampling. Lastly, an explicit measure of sexual harassment was not included even though it is an important component of harassment.

## Conclusion

The main finding of this study is that harassment continues to be a problem for medical students when on clinical placements. In addition, medical students experience harassment in healthcare workplaces, which are their learning settings. These harassment behaviours, self-reported first-hand by medical students, which we consider to be harassment sentinel events, involve experiences of disrespect, exclusion, physical aggression, threats, and verbal mistreatment. The findings indicate that further initiatives need to be designed to identify and respond to workplace harassment, and these initiatives need to occur across all levels of training with all key stakeholders (i.e. teachers, administrators, service personnel, and students). These initiatives may include education of individuals in reference to professional behaviour, boundaries, and communication, or it may require different sorts of reporting mechanisms that are clearly linked to human resource processes or those designed to tackle unprofessional conduct embedded in the workplace culture. Moreover, students need to feel safe when reporting harassment experiences to allow managers and educators to address the full extent of the problem.

## Data Availability

The anonymised data that supports the findings of this study are openly available in the Figshare repository, https://figshare.com/articles/dataset/Medical_students_quality_of_life_and_its_association_with_harassment_and_social_support/14776692
